# Eating problems in ADHD: self-regulatory or inattentive/impulsive

**DOI:** 10.1192/j.eurpsy.2022.1178

**Published:** 2022-09-01

**Authors:** A. Araújo, M. Batista, M.D. Pascoal, A.T. Pereira, F. Ventura, N. Madeira, A. Macedo

**Affiliations:** 1 Centro Hospitalar e Universitário de Coimbra, Department Of Psychiatry, Coimbra, Portugal; 2 Faculty of Medicine of University of Coimbra, Institute Of Psychological Medicine, Coimbra, Portugal; 3 Coimbra Institute for Biomedical Imaging and Translational Research, -, Coimbra, Portugal

**Keywords:** Impulsivity, eating problems, adhd

## Abstract

**Introduction:**

ADHD is a risk factor for impulsive/compulsive eating problems (EP). In, bulimia nervosa and compulsive eating disorder, EP are frequently preceded by negative affect and experienced as loss of control. Clarifying the underlying causes (eg., ADHD symptoms and/or psychological distress) of EP in ADHD would allow the development of targeted interventions.

**Objectives:**

To a) compare levels of EP between ADHD patients and a community sample, and b) test if ADHD symptoms and psychological distress predict EP, in ADHD patients.

**Methods:**

Adults with ADHD (n=32; age=23.78+/-6.12; 69% males) from the Neurodevelopmental Outpatient Unit of Coimbra and healthy participants (n=30; age=36.90+/-13.23; 57% males) answered an online survey including the Portuguese versions of the Adult ADHD Self-Report Scale Symptom Checklist, the Parkinson’s Disease Impulsive-Compulsive Disorders Questionnaire-Current Short and the Depression, Anxiety and Stress Scale.

**Results:**

The ADHD group reported experiencing more EP than healthy individuals (18/32 vs. 4/30; χ2=12.458, p<.001). ADHD patients with EP suffered from severer ADHD inattentive, hyperactive, and global symptoms and higher levels of psychological distress (p<.001 to p=.027). Logistic regression model testing if ADHD and psychological distress symptoms predicted EP, in ADHD, explained 38.8% of the variance and showed that the only significant predictor was ADHD symptoms (B=.121, SE=.051, p=.017).

**Conclusions:**

Our results indicate that EP are associated with severer ADHD clinical pictures. EP arose secondarily to ADHD symptoms, instead of serving as means to alleviate psychological distress. Clinicians should be mindful that, in ADHD patients, EP follow specific motivations, i.e., impulsivity and inattention, and may respond to combined cognitive-behavioural/executive training strategy.

**Disclosure:**

No significant relationships.

